# Early postoperative ipsilateral middle cerebral artery pulsatility index is associated with cerebral hemorrhage after endovascular treatment in anterior circulation large vessel occlusion

**DOI:** 10.3389/fneur.2026.1972755

**Published:** 2026-10-26

**Authors:** Wensheng Zhang, Weiwei Liu, Jumei Zhu, Weifang Xing, Guiping Yang, Hongli Gu, Yunqiang Huang, Minzhen Zhu, Wenjie Zi, Xiaojing Zhong, Guanghong Zhong, Zhenqin Jiang

**Affiliations:** 1 Department of Neurology, Heyuan People’s Hospital, Guangdong Provincial People’s Hospital Heyuan Hospital, Heyuan, Guangdong, China; 2 Heyuan Key Laboratory of Molecular Diagnosis & Disease Prevention and Treatment, Doctors Station of Guangdong Province, Heyuan People’s Hospital, Heyuan, Guangdong, China; 3 Department of Neurology, Xinqiao Hospital and The Second Affiliated Hospital, Army Medical University (Third Military Medical University), Chongqing, China

**Keywords:** acute ischemic stroke, anterior circulation large vessel occlusion, cerebral hemorrhage, endovascular treatment, pulsatility index, transcranial Doppler

## Abstract

**Objective:**

To investigate the predictive value of the early postoperative ipsilateral middle cerebral artery pulsatility index (PI) for cerebral hemorrhage after endovascular treatment in patients with anterior circulation large vessel occlusion acute ischemic stroke.

**Methods:**

A total of 120 patients were divided into a cerebral hemorrhage group (*n* = 17) and a non-hemorrhage group (*n* = 103). Multivariable logistic regression and Firth penalized likelihood regression were used to identify independent predictors. Receiver operating characteristic (ROC) curves evaluated the predictive performance of individual indicators and a combined model. Bootstrap resampling (1,000 iterations) was performed for internal validation.

**Results:**

Multivariable logistic regression showed that diabetes mellitus (OR = 4.154, 95% CI: 1.255–13.756, *p* = 0.020), ASPECTS >8 (OR = 0.115, 95% CI: 0.014–0.942, *p* = 0.044), and elevated ipsilateral PI (OR = 11.355, 95% CI: 1.267–101.761, *p* = 0.030) were associated with cerebral hemorrhage. Firth regression confirmed these results. The ipsilateral PI predicted cerebral hemorrhage with an AUC of 0.716 (95% CI: 0.597–0.835), an optimal cutoff of 0.735, a sensitivity of 0.882, and a specificity of 0.505. The combined model (diabetes, ASPECTS >8, PI) achieved an AUC of 0.808 (95% CI: 0.718–0.899), with a sensitivity of 0.882 and a specificity of 0.650. Bootstrap internal validation showed a bias-corrected AUC of 0.810 (95% CI: 0.717–0.896) and an optimism of −0.0022.

**Conclusion:**

The early postoperative ipsilateral middle cerebral artery PI is a potential prognostic marker for any radiographic intracranial hemorrhage after endovascular treatment in anterior circulation large vessel occlusion acute ischemic stroke, with an optimal cutoff of 0.735. In this cohort, adding PI to diabetes mellitus and ASPECTS was associated with a modest improvement in discrimination (ΔAUC = 0.088, *p* = 0.011) and continuous net reclassification improvement (NRI = 0.594, *p* = 0.020), whereas categorical NRI was not significant. These findings are exploratory and require validation in larger cohorts.

## 1 Introduction

Acute ischemic stroke (AIS) is one of the leading causes of death and disability among adults in China. Anterior circulation large vessel occlusion accounts for approximately 30%–40% of ischemic strokes and is associated with an extremely poor natural prognosis (1, 2). In recent years, endovascular treatment (EVT) has significantly improved the recanalization rate and favorable functional outcomes in large vessel occlusion stroke, and has become a first-line treatment recommended by domestic and international guidelines (3, 4). However, post-procedural cerebral hemorrhagic transformation is one of the most serious complications, occurring in 10%–30% of patients, and symptomatic intracranial hemorrhage can lead to rapid neurological deterioration, significantly increasing mortality and poor prognosis (5, 6). The incidence of asymptomatic intracranial hemorrhage after EVT can be as high as 33%, and asymptomatic intracranial hemorrhage is negatively correlated with functional outcome at follow-up (7). Therefore, early identification of high-risk patients for cerebral hemorrhage and implementation of individualized prevention strategies are of great clinical importance.

Currently, predictors of hemorrhagic transformation are mainly categorized into clinical factors (e.g., advanced age, hyperglycemia, atrial fibrillation), imaging factors (e.g., low ASPECTS, poor collateral circulation), and blood biomarkers (e.g., matrix metalloproteinase-9, cellular fibronectin) (8–12). However, most of these indicators are pre-procedural or static parameters and cannot reflect the dynamic changes of cerebral hemodynamics and microvascular integrity after reperfusion. Transcranial Doppler (TCD) is non-invasive, bedside-applicable, and repeatable (13), allowing immediate assessment of cerebral blood flow velocity and the pulsatility index (PI). PI reflects the resistance status of the cerebrovascular bed; an elevated PI suggests increased distal microvascular resistance or decreased vascular compliance (14, 15). Previous studies have shown associations between TCD parameters and blood–brain barrier disruption or poor outcomes in AIS patients (16–18), but the value of the early postoperative ipsilateral Middle cerebral artery (MCA) PI for predicting cerebral hemorrhage after EVT has not been systematically studied.

While the association between abnormal cerebral hemodynamics and adverse post-reperfusion outcomes has been previously explored, whether early postoperative PI provides incremental predictive value beyond diabetes mellitus and ASPECTS, both selected from the present dataset, has not been systematically evaluated. Therefore, we retrospectively analyzed clinical data from 120 patients with anterior circulation large vessel occlusion AIS who underwent EVT, to assess whether early postoperative ipsilateral MCA PI adds clinically useful predictive information beyond diabetes mellitus and ASPECTS.

## 2 Materials and methods

### 2.1 Study population

Consecutive patients with anterior circulation large vessel occlusion AIS admitted to Heyuan People’s Hospital from March 2021 to May 2022 were enrolled. Inclusion criteria: (1) age ≥18 years; (2) confirmation of internal carotid artery, M1 segment of the MCA, or M2-segment occlusion by CT angiography (CTA) or magnetic resonance angiography (MRA); (3) time from onset to femoral artery puncture ≤24 h; (4) successful recanalization with post-procedural expanded Thrombolysis in Cerebral Infarction (eTICI) grade ≥2b; (5) early postoperative TCD examination completed. Exclusion criteria: (1) posterior circulation large vessel occlusion AIS; (2) confirmed pre-procedural hemorrhagic transformation; (3) inability to complete early postoperative TCD examination.

A total of 120 patients were enrolled, including 78 males (65.00%) and 42 females (35.00%), with a mean age of 67.00 ± 11.20 years.

### 2.2 Endovascular treatment and post-procedural management

All patients underwent EVT under local or general anesthesia. Based on the occlusion site and thrombus burden, stent retriever thrombectomy, aspiration thrombectomy, or a combination of both was used. Immediate post-procedural digital subtraction angiography (DSA) was performed to assess recanalization using the eTICI grading system: grade 2b (50%–89% perfusion of the distal ischemic territory), grade 2c (90%–99%), and grade 3 (complete recanalization).

Post-procedural management followed the guidelines for EVT of acute ischemic stroke (4, 19). For patients who received intravenous thrombolysis, antiplatelet therapy was initiated after a CT scan within 24 h following thrombolysis, based on the CT findings.

### 2.3 Transcranial Doppler examination

All patients underwent early postoperative TCD examination (within 6 h after EVT). A Delica EMS-9D TCD device with a 2 MHz pulsed-wave probe was used. The examination was performed by one neuro-sonographer with >5 years of TCD experience, and the average of three measurements was recorded. The TCD operator was blinded to subsequent imaging outcomes, as the follow-up CT scan was performed at 24 h after the procedure or later, whereas TCD was performed within 6 h.

The temporal window was routinely used to obtain flow signals from the bilateral MCA M1 segments; if the temporal window was insufficient, the orbital window was used. The following flow parameters were recorded: systolic peak velocity (*Vs*), end-diastolic velocity (Vd), mean velocity (Vm), and pulsatility index (PI). PI was calculated as PI = (*Vs* – Vd)/Vm. The ipsilateral side was defined as the side of pre-procedural vessel occlusion, and the contralateral side as the opposite side. All measurements were obtained under quiet breathing, and for each side at least three stable spectral waveforms were averaged.

All measurements were performed by a single experienced operator to minimize inter-observer variability, and the average of three consecutive stable waveforms was used to enhance reliability. Intra-observer reliability was not formally assessed; however, the average of three consecutive stable waveforms was used to enhance reliability.

### 2.4 Diagnosis and grouping of cerebral hemorrhage

A non-contrast head CT scan was routinely performed 24 h after EVT. An additional urgent CT scan was performed earlier in patients with neurological deterioration, defined as an increase of ≥4 points in the NIHSS score. A routine immediate postoperative cranial CT was performed in all patients before TCD examination, and no definite hemorrhage was identified on this immediate postoperative CT. However, because residual contrast medium can produce hyperdense signals that mimic or obscure hemorrhage, this immediate CT could not reliably exclude a small or obscured pre-existing hemorrhage. Therefore, the diagnosis of cerebral hemorrhage was based on the follow-up CT at 24 h, or on an urgent CT performed earlier only in patients with neurological deterioration. No patient had documented neurological deterioration before TCD examination. Accordingly, pre-TCD hemorrhage could not be definitively excluded.

Cerebral hemorrhage was defined as the appearance of a new hyperdense lesion on CT, diagnosed according to the Heidelberg Bleeding Classification (20, 21). Hemorrhagic transformation was further categorized as hemorrhagic infarction (HI-1 or HI-2), parenchymal hematoma (PH-1 or PH-2), or intraventricular hemorrhage (IVH), according to the Heidelberg Bleeding Classification. Symptomatic intracranial hemorrhage (sICH) was defined as new intracranial hemorrhage on imaging accompanied by an increase of ≥4 points in the National Institutes of Health Stroke Scale (NIHSS) score or leading to death or surgery; asymptomatic intracranial hemorrhage (aICH) was defined as radiographic hemorrhage without such neurological deterioration. The primary outcome was any radiographic intracranial hemorrhage (symptomatic or asymptomatic). Patients were divided into a cerebral hemorrhage group (*n* = 17) and a non-hemorrhage group (*n* = 103).

### 2.5 Clinical data collection

Baseline data collected included: (1) general information: sex, age; (2) vascular risk factors: hypertension, diabetes mellitus, coronary heart disease, atrial fibrillation, prior stroke, smoking; (3) clinical assessments: pre-stroke modified Rankin Scale (mRS) score, admission NIHSS score (categorized as 6–15 and >15), Alberta Stroke Program Early CT Score (ASPECTS); (4) treatment-related data: receipt of intravenous thrombolysis, occlusion site, collateral circulation grade (ASITN/SIR grading system, 0–4; good collateral circulation defined as grade ≥3), recanalization grade (eTICI); (5) time metrics: onset-to-door time, door-to-puncture time, puncture-to-recanalization time; (6) stroke mechanism: large artery atherosclerosis, cardioembolism, other determined causes. Data on prior antiplatelet or anticoagulant use, dyslipidemia, and chronic kidney disease were not systematically available in the retrospective database; therefore, these variables could not be included in the analysis.

### 2.6 Statistical analysis

Statistical analyses were performed using SPSS 26.0 and R 4.5.2. Normally distributed continuous data are expressed as mean ± standard deviation (SD) and were compared using the independent samples t-test. Non-normally distributed continuous data are expressed as median (interquartile range, IQR) and were compared using the Mann–Whitney U test. Categorical data are expressed as counts (percentages) and were compared using the *χ*^2^ test or Fisher’s exact test.

ASPECTS was dichotomized as ≤8 versus >8, consistent with prior studies (22, 23), given its clinical relevance for risk stratification, and because treating it as continuous would have consumed excessive degrees of freedom with only 17 hemorrhagic events.

Variables with *p* < 0.05 in univariate analysis were entered into a multivariable logistic regression model (forward stepwise) to calculate odds ratios (OR) with 95% confidence intervals (CI). Receiver operating characteristic (ROC) curves were used to evaluate the predictive performance of individual indicators and the combined model; the area under the curve (AUC), sensitivity, specificity, and optimal cutoff (maximizing the Youden index) were calculated. The AUCs of different models were compared using the DeLong test, and a two-sided *p* < 0.05 was considered statistically significant. To address potential small-sample bias due to the limited number of events (*n* = 17), Firth penalized likelihood regression was performed as a sensitivity analysis; this method reduces bias in maximum likelihood estimation by applying a penalty to the likelihood function, and is particularly suitable when the number of events per variable is low.

Bootstrap resampling with 1,000 iterations was used for internal validation of the combined model. In each iteration, a bootstrap sample was drawn with replacement from the original dataset; the model was refitted in the bootstrap sample, and its AUC was calculated both in the bootstrap sample (apparent performance) and in the original dataset (test performance). The optimism was defined as the difference between the apparent and test AUC. The mean optimism across 1,000 iterations was subtracted from the original AUC to obtain the optimism-corrected AUC. Calibration slope, intercept, and Brier score were also obtained from the bootstrap procedure. Given the limited number of events in this exploratory study, these internal validation results should be interpreted as exploratory and do not establish definitive model stability. A two-sided *p* < 0.05 was considered statistically significant.

To assess the incremental predictive value of PI, a reference model including diabetes mellitus and ASPECTS was compared with an extended model additionally including PI. Model performance was evaluated by ΔAUC (DeLong test), likelihood-ratio test, continuous and categorical net reclassification improvement (NRI), integrated discrimination improvement (IDI), calibration, Brier score, and decision curve analysis.

Missing data were assessed for all candidate variables; no missing values were present. Complete-case analysis was used, and no imputation was performed.

## 3 Results

### 3.1 Study flowchart

A total of 186 patients who underwent EVT were initially assessed. After excluding 66 patients, 120 patients with anterior circulation large vessel occlusion AIS were included in the final analysis. The study flowchart is shown in Figure 1.

**Figure 1:**
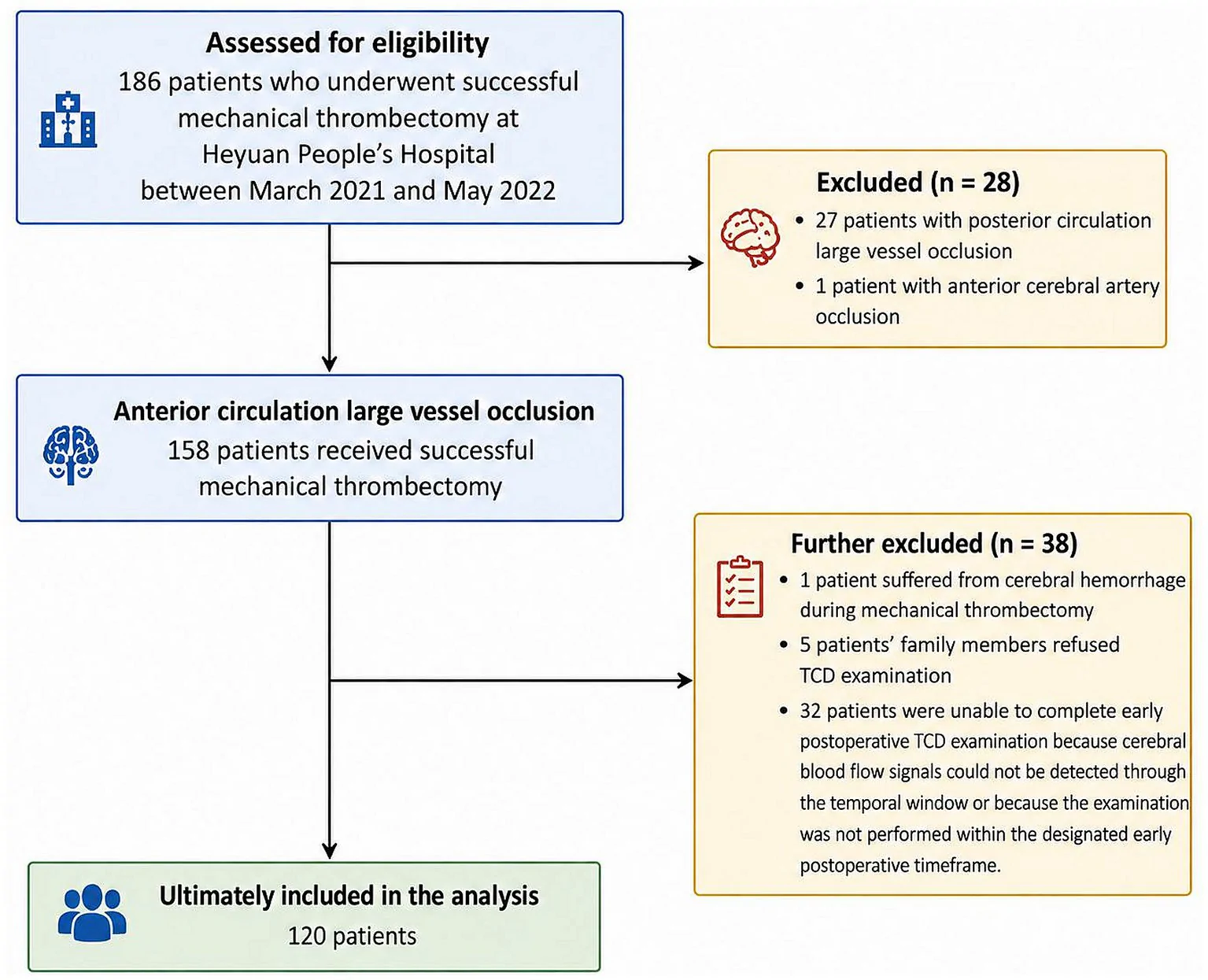
Study flowchart. A total of 186 patients who underwent successful EVT at Heyuan People’s Hospital from March 2021 to May 2022 were initially assessed. Twenty-eight patients were excluded due to posterior circulation large vessel occlusion (*n* = 27) or anterior cerebral artery occlusion (*n* = 1). Of the remaining 158 patients with anterior circulation large vessel occlusion, 38 were further excluded because of intracranial hemorrhage during the procedure (*n* = 1), refusal by family members to undergo TCD examination (*n* = 5), or inability to complete early postoperative TCD examination due to an inadequate temporal acoustic window (*n* = 32). Finally, 120 patients were included in the analysis and divided into hemorrhage group (*n* = 17) and non-hemorrhage group (*n* = 103). Note: All patients underwent a routine immediate postoperative cranial CT before TCD examination; however, because residual contrast medium can produce hyperdense signals, this CT could not reliably exclude hemorrhage at that time. No patient had documented neurological deterioration before TCD. Therefore, pre-existing hemorrhage had not been definitively excluded by imaging before TCD.

### 3.2 Baseline characteristics

Among the 120 patients, 17 (14.17%) developed cerebral hemorrhage. As shown in Table 1, there were no significant differences between the hemorrhage and non-hemorrhage groups in sex, age, hypertension, coronary heart disease, atrial fibrillation, prior stroke, smoking, pre-stroke mRS, NIHSS categories, intravenous thrombolysis, occlusion site, good collateral circulation (grade ≥3), eTICI grade, or time metrics (all *p* > 0.05). The hemorrhage group had a significantly higher proportion of diabetes mellitus (41.18% vs. 13.59%, *p* = 0.006) and a significantly lower proportion of ASPECTS >8 (5.88% vs. 33.98%, *p* = 0.019) compared to the non-hemorrhage group.

**Table 1:** Baseline clinical characteristics of patients with and without cerebral hemorrhage after endovascular treatment for acute ischemic stroke due to anterior circulation large vessel occlusion.

Variable	All patients (*n* = 120)	Hemorrhage (*n* = 17)	No hemorrhage (*n* = 103)	*p*-value
General information				
Male sex, *n* (%)	78 (65.00)	12 (70.59)	66 (64.08)	0.602
Age (years), mean ± SD	67.00 ± 11.20	67.00 ± 9.68	67.00 ± 11.46	0.871
Medical history				
Hypertension, *n* (%)	73 (60.83)	13 (76.47)	60 (58.25)	0.154
Diabetes mellitus, *n* (%)	21 (17.50)	7 (41.18)	14 (13.59)	**0.006**
Coronary heart disease, *n* (%)	19 (15.83)	3 (17.65)	16 (15.53)	0.825
Atrial fibrillation, *n* (%)	21 (17.50)	2 (11.76)	19 (18.45)	0.502
Prior stroke, *n* (%)	20 (16.67)	2 (11.76)	18 (17.48)	0.558
Smoking, *n* (%)	45 (37.50)	7 (41.18)	38 (36.89)	0.735
Pre-stroke mRS score, mean ± SD	0.183 ± 0.534	0.294 ± 0.686	0.165 ± 0.507	0.395
Stroke presentation				
NIHSS score, *n* (%)				0.437
6–15	87 (72.50)	11 (64.71)	76 (73.79)	
>15	33 (27.50)	6 (35.29)	27 (26.21)	
ASPECTS >8, *n* (%)	36(30.00)	1 (5.88)	35(33.98)	**0.019**
Intravenous thrombolysis, *n* (%)	41 (34.17)	4 (23.53)	37 (35.92)	0.318
Occlusion location, *n* (%)				0.149
Middle cerebral artery	79 (65.83)	8 (47.06)	71 (68.93)	
Internal carotid artery	31 (25.83)	6 (35.29)	25 (24.27)	
Anterior circulation tandem lesion	10 (8.33)	3 (17.65)	7 (6.79)	
Good collateral circulation*, *n* (%)	11 (9.17)	0 (0)	11 (10.68)	0.157
Recanalization grade (eTICI), *n* (%)				0.684
2b	31 (25.83)	5 (29.41)	26 (25.24)	
2c	4 (3.33)	0 (0)	4 (3.88)	
3	85 (70.83)	12 (70.59)	73 (70.87)	
Time metrics				
Onset-to-door time (min), median (IQR)	360.00 (240.00–540.00)	360.00 (240.00–570.00)	360.00 (240.00–540.00)	0.988
Door-to-puncture time (min), median (IQR)	116.50 (96.25–143.00)	116.00 (92.50–128.00)	117.00 (98.00–144.00)	0.306
Puncture-to-recanalization time (min), median (IQR)	98.50 (80.00–124.50)	96.00 (73.50–120.50)	99.00 (80.00–125.00)	0.830
Stroke mechanism, n (%)				0.345
Large artery atherosclerosis	74 (61.67)	11 (64.71)	63 (61.17)	
Cardioembolism	28 (23.33)	2 (11.76)	26 (25.24)	
Dissection	18 (15.00)	4 (23.53)	14 (13.59)	
Post-procedural TCD flow parameters				
Ipsilateral *Vs* (cm/s), median (IQR)	83.00 (67.25–112.75)	94.00 (67.00–114.50)	82.00 (67.00–113.00)	0.880
Ipsilateral Vd (cm/s), median (IQR)	40.50 (33.13–53.50)	37.00 (31.75–56.25)	40.50 (33.50–53.50)	0.479
Ipsilateral Vm (cm/s), median (IQR)	53.50 (45.00–73.75)	55.00 (43.50–76.00)	53.00 (46.00–74.00)	0.251
Ipsilateral PI	0.75 (0.63–0.92)	0.91 (0.75–1.06)	0.73 (0.62–0.88)	**0.004**
Contralateral *Vs* (cm/s), median (IQR)	86.00 (77.00–107.00)	85.00 (79.00–104.50)	86.00 (76.00–108.00)	0.311
Contralateral Vd (cm/s), median (IQR)	42.25 (36.25–50.38)	40.50 (31.75–47.00)	42.50 (37.50–50.50)	0.102
Contralateral Vm (cm/s), median (IQR)	57.00 (51.25–68.00)	54.00 (48.00–64.50)	58.00 (52.00–68.00)	0.324
Contralateral PI	1.01 (0.83–1.15)	1.08 (0.83–1.11)	1.00 (0.83–1.15)	0.830

ASPECTS = Alberta stroke program early CT score; eTICI = expanded thrombolysis in cerebral infarction; TCD = transcranial Doppler; Vs = systolic peak velocity; Vd = end-diastolic velocity; Vm = mean velocity; PI = pulsatility index. *Good collateral circulation was defined as ASITN/SIR grade ≥3 (scale 0–4). Bold values indicate statistical significance at *p* < 0.05.

As shown in Table 2, among the 17 patients with radiographic intracranial hemorrhage, 5 had symptomatic intracranial hemorrhage and 12 had asymptomatic intracranial hemorrhage. According to the Heidelberg Bleeding Classification, 8 patients had hemorrhagic infarction (HI-1, *n* = 3; HI-2, *n* = 5), 8 had parenchymal hematoma (PH-1, *n* = 4; PH-2, *n* = 4), and 1 had intraventricular hemorrhage. Due to the small number of events in each subtype, separate multivariable analyses for symptomatic ICH, PH, IVH, or other subtypes were not feasible.

**Table 2:** Subtypes of intracranial hemorrhage after endovascular treatment (*n* = 17).

Hemorrhage subtype	*n*
Symptomatic ICH	5
Asymptomatic ICH	12
HI-1	3
HI-2	5
PH-1	4
PH-2	4
Intraventricular hemorrhage	1
Total	**17**

HI = hemorrhagic infarction; PH = parenchymal hematoma; IVH = intraventricular hemorrhage; ICH = intracranial hemorrhage. Subtypes were classified according to the Heidelberg Bleeding Classification. Symptomatic status and imaging subtype are separate dimensions. All 17 hemorrhagic events were reliably classified after re-review of imaging. Bold values indicate statistical significance at *p* < 0.05.

### 3.3 Early postoperative TCD flow parameters

The ipsilateral MCA PI was significantly higher in the hemorrhage group than in the non-hemorrhage group [0.91 (0.75–1.06) vs. 0.73 (0.62–0.88), *p* = 0.004]. No significant differences were observed in ipsilateral *Vs*, Vd, Vm, or any contralateral parameters (all *p* > 0.05). The TCD parameters are presented in Table 1.

### 3.4 Multivariable logistic regression for independent predictors

Variables with *p* < 0.05 in univariate analysis (diabetes mellitus, ASPECTS >8, ipsilateral PI) were entered into the multivariable logistic regression model. Results showed that diabetes mellitus (OR = 4.154, 95% CI: 1.255–13.756, *p* = 0.020) and elevated ipsilateral PI (OR = 11.355, 95% CI: 1.267–101.761, *p* = 0.030) were associated with cerebral hemorrhage, while ASPECTS >8 (OR = 0.115, 95% CI: 0.014–0.942, *p* = 0.044) was inversely associated with cerebral hemorrhage. However, the wide confidence intervals for these estimates, particularly for PI, indicate substantial uncertainty due to the limited number of hemorrhagic events (n = 17). The multivariable model should therefore be regarded as exploratory, and the effect sizes should not be overinterpreted. The multivariable logistic regression results are presented in Table 3, and the forest plot for the ipsilateral MCA PI is shown in Figure 2.

**Table 3:** Univariate and Multivariable Logistic Regression Analysis for Cerebral Hemorrhage.

Variable	Univariate analysis			Multivariate analysis		
	OR	95% CI	*p*-value	OR	95% CI	*p*-value
Diabetes	4.450	1.454–13.615	**0.009**	4.154	1.255–13.756	**0.020**
ASPECTS >8	0.121	0.015–0.954	**0.045**	0.115	0.014–0.942	**0.044**
PI value of the responsible middle cerebral artery	11.743	1.461–94.391	**0.021**	11.355	1.267–101.761	**0.030**

MCA = middle cerebral artery; PI = pulsatility index. Bold values indicate statistical significance at *p* < 0.05.

**Figure 2:**
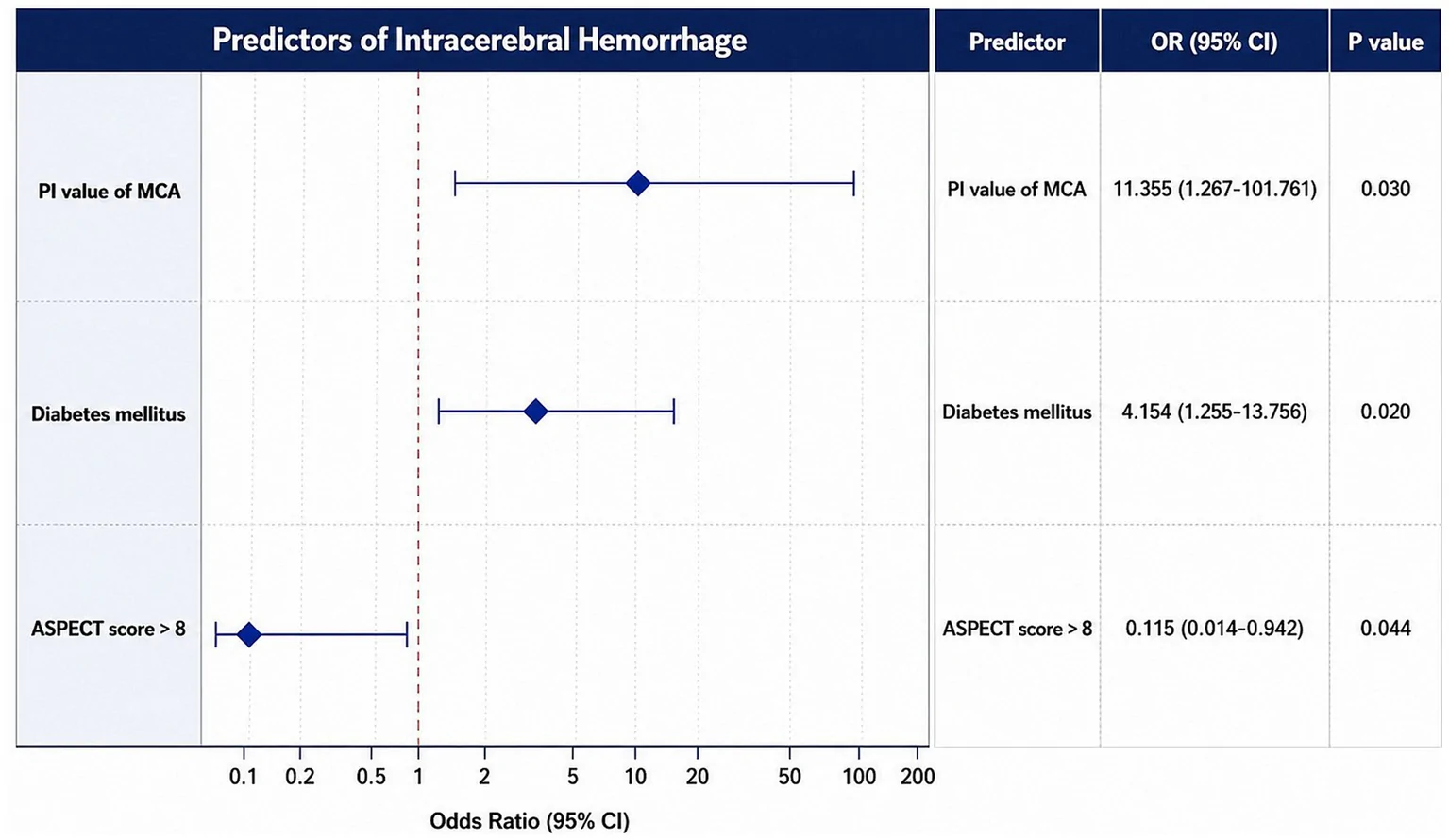
Forest plot of independent predictors of cerebral hemorrhage after endovascular treatment (EVT). The forest plot displays the odds ratios (ORs) with their 95% confidence intervals (CIs) on a logarithmic scale. The dashed vertical line indicates OR = 1 (null effect). Squares represent the weight of each factor, and horizontal lines represent the 95% CI. Ipsilateral MCA PI value (OR = 11.36, 95% CI: 1.27–101.76, *p* = 0.030) and diabetes mellitus (OR = 4.15, 95% CI: 1.25–13.76, *p* = 0.020) were associated with cerebral hemorrhage, while ASPECTS >8 (OR = 0.12, 95% CI: 0.01–0.94, *p* = 0.044) was inversely associated with cerebral hemorrhage.

### 3.5 Firth penalized likelihood regression sensitivity analysis

To address potential bias due to the small sample size, we performed Firth regression as a sensitivity analysis. Results (Table 4) showed that diabetes mellitus (OR = 3.661, 95% CI: 1.150–11.543, *p* = 0.029), ASPECTS >8 (OR = 0.176, 95% CI: 0.018–0.779, *p* = 0.019), and ipsilateral PI (OR = 9.869, 95% CI: 1.370–97.345, *p* = 0.024) remained significantly associated with cerebral hemorrhage, with effect directions consistent with the conventional logistic regression. The OR estimates were slightly attenuated and the confidence intervals were somewhat narrower, suggesting that the conventional model was not substantially biased. For the same reason, this model should also be regarded as exploratory.

**Table 4:** Firth penalized likelihood regression (sensitivity analysis) for cerebral hemorrhage.

Variable	OR	95% CI	*p*-value
Diabetes mellitus	3.661	1.150–11.543	**0.029**
ASPECTS >8	0.176	0.018–0.779	**0.019**
Ipsilateral MCA PI value	9.869	1.370–97.345	**0.024**

Firth regression was used to reduce bias due to small sample size (only 17 hemorrhage events). Bold values indicate statistical significance at *p* < 0.05.

### 3.6 ROC curve analysis of individual indicators and the combined model

ROC curve analysis (Figure 3) showed that the ipsilateral MCA PI predicted cerebral hemorrhage with an AUC of 0.716 (95% CI: 0.597–0.835), an optimal cutoff of 0.735 (Youden index 0.387), a sensitivity of 0.882, and a specificity of 0.505. Diabetes alone had an AUC of 0.633 (95% CI: 0.506–0.756), and ASPECTS ≤8 had an AUC of 0.640 (95% CI: 0.557–0.709). The combined model of the three indicators achieved an AUC of 0.808 (95% CI: 0.718–0.899), with a sensitivity of 0.882 and a specificity of 0.650, significantly outperforming each single indicator by the DeLong test (*p* < 0.05).

**Figure 3:**
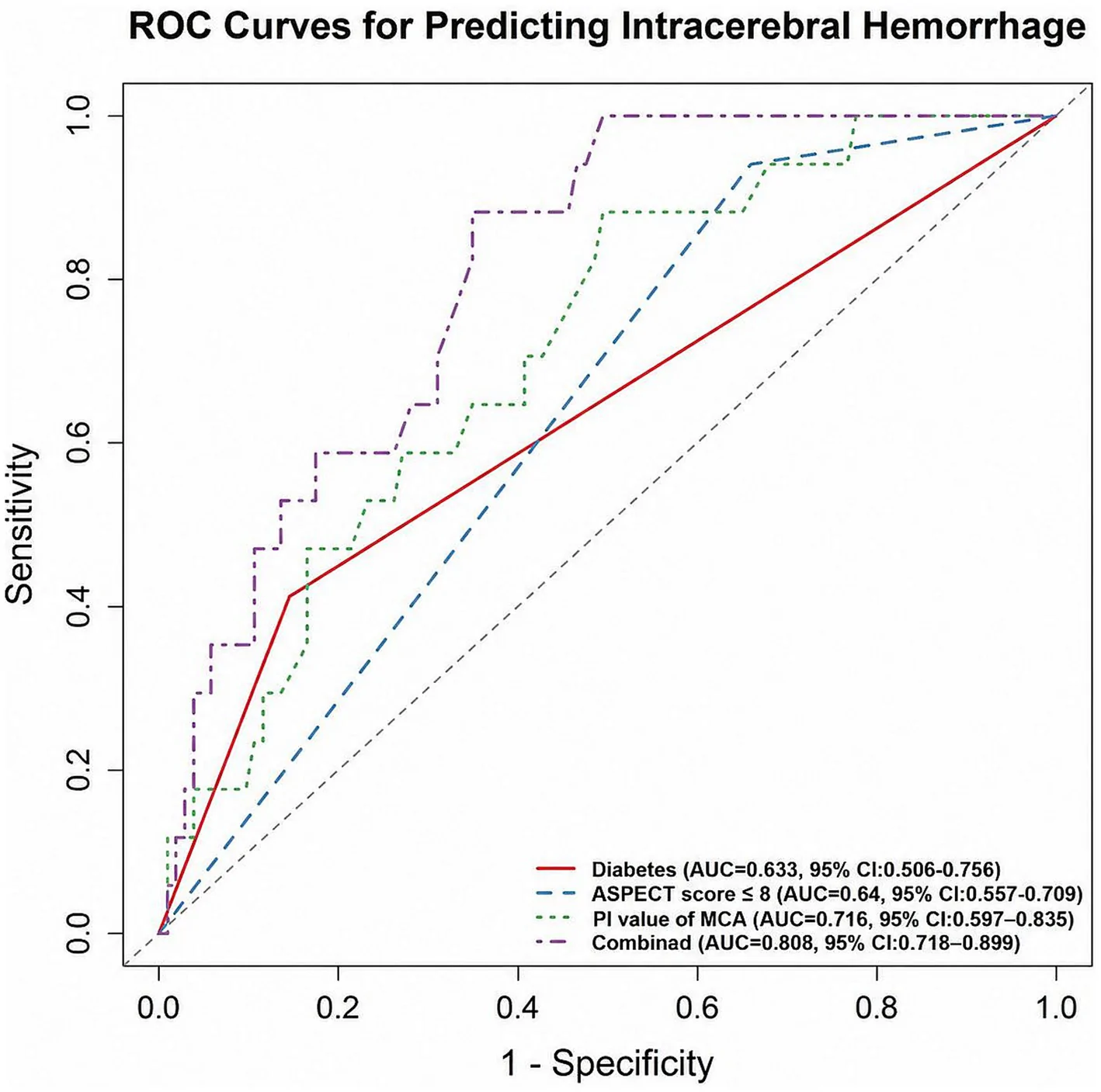
ROC curves of individual predictors and the combined model for cerebral hemorrhage. ROC curves showing the predictive performance of ipsilateral MCA PI value (AUC = 0.716, 95% CI: 0.597–0.835), diabetes mellitus (AUC = 0.633, 95% CI: 0.506–0.756), ASPECTS ≤8 (AUC = 0.640, 95% CI: 0.557–0.709), and the combined model (AUC = 0.808, 95% CI: 0.718–0.899). The combined model significantly outperformed each single indicator (*p* < 0.05).

In the incremental value analysis, the reference model (diabetes + ASPECTS) achieved an AUC of 0.720 (95% CI: 0.618–0.822), whereas the extended model including PI achieved an AUC of 0.808 (95% CI: 0.717–0.900), with a ΔAUC of 0.088 (95% CI: 0.021–0.156; *p* = 0.011 by DeLong test). The likelihood-ratio test was significant (*χ*^2^ = 5.003, df = 1, *p* = 0.025). The continuous NRI was 0.594 (95% CI: 0.094–1.094; *p* = 0.020) and the IDI was 0.050 (95% CI: 0.001–0.099; *p* = 0.044), whereas the categorical NRI was of borderline significance (0.216, 95% CI: −0.006–0.439; *p* = 0.057). Calibration of the extended model was good (slope = 1.00, intercept ≈ 0, Brier score = 0.1045). Decision curve analysis showed a modest net benefit for the extended model across a threshold probability range of approximately 10%–30% (Figure 4). These results are summarized in Table 5.

**Figure 4:**
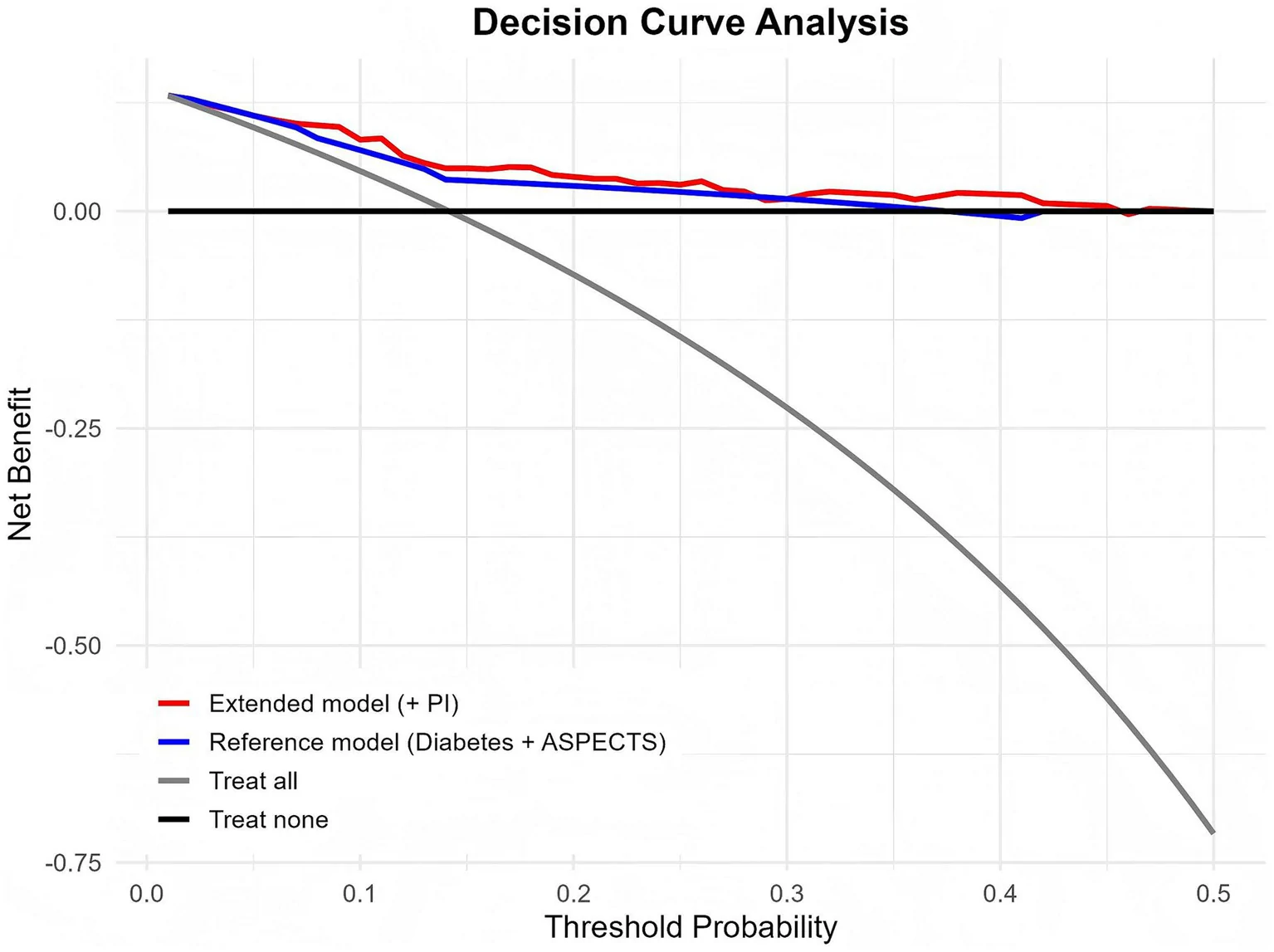
Decision curve analysis comparing the reference model and the extended model. Decision curve analysis showing the net benefit of the reference model (diabetes + ASPECTS) and the extended model (diabetes + ASPECTS + PI) across a range of threshold probabilities. The extended model provided a modest net benefit compared with the reference model across a clinically relevant threshold probability range of approximately 10%–30%. Beyond this range, the two models performed similarly. The gray line represents “treat all,” and the black line represents “treat none”.

**Table 5:** Incremental predictive value of PI for cerebral hemorrhage after endovascular treatment.

Model	AUC (95% CI)	ΔAUC (95% CI)	P (DeLong)	NRI (continuous, 95% CI)	P	IDI (95% CI)	P	Brier score
Reference (diabetes + ASPECTS)	0.720 (0.618–0.822)	–	–	–	–	–	–	0.1099
Extended (+ PI)	0.808 (0.717–0.900)	0.088 (0.021–0.156)	0.011	0.594 (0.094–1.094)	0.020	0.050 (0.001–0.099)	0.044	0.1045

AUC = area under the receiver operating characteristic curve; CI = confidence interval; NRI = net reclassification improvement; IDI = integrated discrimination improvement. ΔAUC = AUC (extended model) – AUC (reference model). *p*-values for ΔAUC were calculated using the DeLong test. The categorical NRI was 0.216 (95% CI: −0.006–0.439; *p* = 0.057).

### 3.7 Bootstrap internal validation and model calibration

Bootstrap resampling (1,000 iterations) for internal validation showed that the original AUC of the combined model was 0.808; the bootstrap mean AUC was 0.810 (95% CI: 0.717–0.896); the optimism was −0.0022; and the bias-corrected AUC was 0.810. These findings suggest that overfitting was not prominent in this dataset. However, given that only 17 hemorrhagic events occurred, these results should be interpreted as exploratory and do not establish definitive model stability. The calibration curve (Figure 5) indicated acceptable calibration in this cohort, with a slope of 1.019, an intercept of −0.003, and a Brier score of 0.1045. Nevertheless, this calibration assessment should also be interpreted cautiously given the limited number of events.

**Figure 5:**
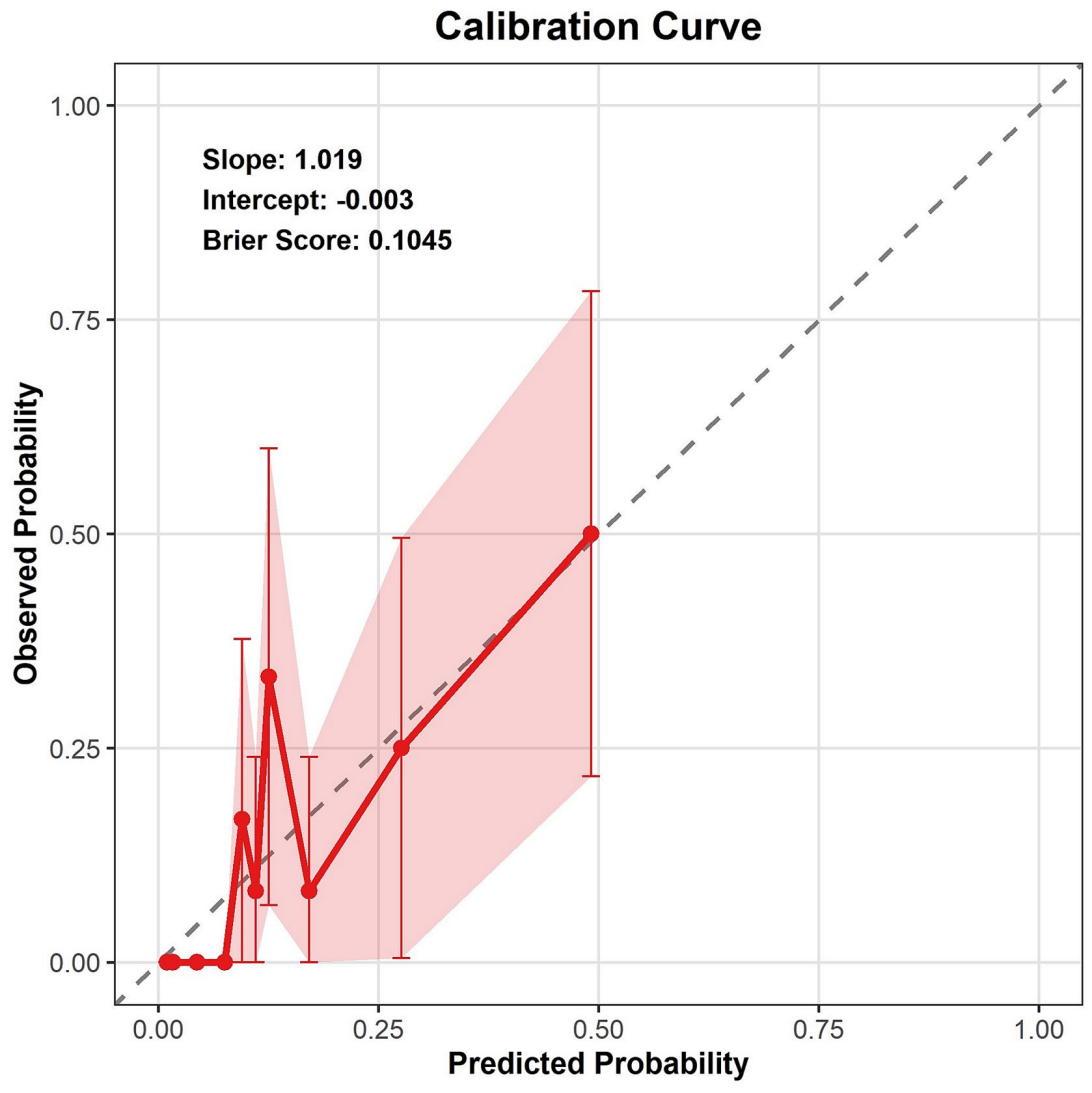
Calibration Curve of the Combined Model. Calibration plot showing predicted vs. observed probability of cerebral hemorrhage. The dashed diagonal line represents perfect prediction. The calibration curve (solid line) closely aligns with the diagonal. Slope = 1.019, Intercept = −0.003, Brier Score = 0.1045, indicating acceptable calibration.

## 4 Discussion

This study investigated the predictive value of early postoperative ipsilateral MCA PI for cerebral hemorrhage after EVT in anterior circulation large vessel occlusion AIS. The main findings are: (1) the ipsilateral MCA PI was independently associated with cerebral hemorrhage in this cohort, with an optimal cutoff of 0.735; (2) the combined model incorporating PI, diabetes mellitus, and ASPECTS showed good discrimination (AUC = 0.808) and showed acceptable internal validation performance; however, given the limited number of events, definitive conclusions about model stability cannot be drawn; (3) Firth regression sensitivity analysis suggested the consistency of the conventional logistic regression results, although the small sample size limits definitive conclusions.

An important limitation of this study is the uncertain temporal relationship between PI measurement and the onset of hemorrhage. PI was measured within 6 h after EVT, whereas hemorrhage was diagnosed on routine CT at 24 h or on immediate CT in patients with neurological deterioration. Because the immediate postoperative CT could not reliably exclude hemorrhage before TCD (due to residual contrast medium producing hyperdense signals), we cannot exclude the possibility that some patients already had occult hemorrhage at the time of PI measurement. Thus, elevated PI may represent an antecedent risk marker, an early physiological manifestation of evolving reperfusion injury, or a concomitant epiphenomenon rather than a causal predictor of hemorrhage initiation. Accordingly, the present study should be regarded as an early post-EVT risk-stratification study rather than a study demonstrating that PI independently predicts the initiation of hemorrhage. Future studies with pre-TCD imaging or serial TCD monitoring are needed to clarify the temporal sequence.

Regarding the primary outcome, the present study adopted any radiographic intracranial hemorrhage (including both symptomatic and asymptomatic) as the primary outcome, rather than symptomatic ICH alone. This choice was based on three considerations. First, asymptomatic ICH is not clinically negligible: a recent multicenter study demonstrated that asymptomatic intracranial hemorrhage after endovascular thrombectomy is independently associated with poorer functional outcomes at follow-up (7). Second, PI elevation may reflect microvascular resistance and blood–brain barrier disruption, which are upstream pathophysiological mechanisms common to all types of hemorrhagic transformation; thus, any ICH serves as a more direct biological endpoint for evaluating microvascular injury. Third, with only 17 hemorrhagic events, restricting the outcome to symptomatic ICH (which occurs in approximately 5%–10% of cases) would yield insufficient statistical power for multivariable analysis. Therefore, the combined model should be interpreted as a potential risk-stratification marker with high sensitivity (0.882) to identify patients at risk of any hemorrhagic event, rather than a tool for predicting clinically overt hemorrhage.

### 4.1 Mechanisms linking high PI to cerebral hemorrhage

PI = (*Vs* – Vd)/Vm is a nonspecific hemodynamic parameter that primarily reflects the resistance and compliance of the distal cerebrovascular bed (14, 15). A high PI after recanalization may indicate increased microcirculatory resistance. Several pathophysiological mechanisms have been hypothesized to contribute to this relationship: ① Ischemia–reperfusion injury, in which prolonged ischemia may damage the blood–brain barrier (BBB) and restored blood flow after recanalization may lead to passive congestion, impaired autoregulation, and elevated distal resistance (13); ② Microvascular dysfunction, in which endothelial swelling, leukocyte adhesion, and no-reflow phenomena may increase microcirculatory resistance (14, 16); ③ Vasogenic edema, in which BBB disruption may allow fluid extravasation into the interstitium, compressing microvessels and further raising resistance (24, 25). These mechanisms are biologically plausible but were not directly measured in this study; BBB permeability, microvascular function, and no-reflow were not assessed, and PI should therefore not be regarded as a validated surrogate of BBB disruption or microvascular injury. While the association between elevated PI and hemorrhagic transformation is supported by our data, the mechanistic explanations remain hypothetical and require direct measurement in future studies. In our study, the median PI in the hemorrhage group was 0.91, significantly higher than the 0.73 in the non-hemorrhage group, and the ROC-determined cutoff of 0.735 is consistent with the lower limit of the normal PI range (approximately 0.6–1.1), indicating good clinical applicability.

### 4.2 Role of diabetes mellitus and ASPECTS

Previous studies have found that a history of diabetes mellitus increases the risk of cerebral hemorrhage after EVT in AIS, possibly related to hyperglycemia-induced basement membrane thickening, endothelial dysfunction, advanced glycation end-product deposition, and oxidative stress that exacerbates reperfusion injury (26–28). An ASPECTS ≤8 indicates a larger infarct core and more severe BBB disruption, whereas a higher ASPECTS (i.e., >8) suggests a smaller infarct core and a lower risk of hemorrhage, consistent with prior reports (29–31). The addition of PI to a reference model containing diabetes mellitus and ASPECTS was associated with improved discrimination (ΔAUC = 0.088, *p* = 0.011), with a continuous NRI of 0.594 and an IDI of 0.050. However, because both variables in the reference model were selected from the present dataset, this finding should be interpreted only relative to this specific reference model, rather than as evidence that PI provides incremental value beyond all established predictors. Given the limited number of events and the borderline categorical NRI, these findings should be regarded as exploratory, and PI should be considered a potential incremental marker rather than an established predictor. No therapeutic recommendations can be made based on these retrospective findings.

### 4.3 Necessity of firth regression and interpretation of results

Because there were only 17 hemorrhagic events, conventional logistic regression might be prone to overfitting or bias. Firth regression reduces small-sample bias through a penalized likelihood function. Its results showed effect directions identical to those of the primary analysis, with some shrinkage in ORs (diabetes 4.15 → 3.66; PI 11.36 → 9.87) but more reasonable CI (PI CI from 1.27–101.76 to 1.37–97.35), and all *p*-values remained <0.05, suggesting the consistency of the primary conclusions, although the small sample size limits definitive conclusions.

### 4.4 Clinical implications and limitations

TCD is non-invasive, bedside-capable, and repeatable. Early postoperative measurement of the ipsilateral PI may serve as a potential risk marker to identify patients who warrant closer monitoring, but it should not be used to guide therapeutic decisions until prospectively validated. Although some clinicians in our center may have adopted more intensive monitoring strategies in patients with higher PI, this was not a protocolized intervention and its efficacy remains unproven. Because of the retrospective design and the limited number of events, no therapeutic recommendations can be made based on these findings.

An important limitation of this study is the uncertain temporal relationship between PI measurement and the onset of hemorrhage. PI was measured within 6 h after EVT, whereas hemorrhage was diagnosed on routine CT at 24 h or on immediate CT in patients with neurological deterioration. Because the immediate postoperative CT could not reliably exclude hemorrhage before TCD (due to residual contrast medium producing hyperdense signals), we cannot exclude the possibility that some patients already had occult hemorrhage at the time of PI measurement. Thus, elevated PI may represent an antecedent risk marker, an early physiological manifestation of evolving reperfusion injury, or a concomitant epiphenomenon rather than a causal predictor of hemorrhage initiation. Accordingly, the present study should be regarded as an early post-EVT risk-stratification study rather than a study demonstrating that PI independently predicts the initiation of hemorrhage. Future studies with pre-TCD imaging or serial TCD monitoring are needed to clarify the temporal sequence.

We acknowledge that combining symptomatic and asymptomatic intracranial hemorrhage into a single composite endpoint is a limitation. These entities differ in clinical severity, prognosis, and therapeutic implications. The composite endpoint was chosen primarily to preserve statistical power and because both phenotypes have been associated with worse outcomes (7). However, this approach may dilute subtype-specific associations and limits the clinical interpretation of our findings. In particular, our model should not be interpreted as predicting clinically overt or symptomatic hemorrhage. Furthermore, although all 17 hemorrhagic events were reliably classified according to the Heidelberg Bleeding Classification, only 5 were symptomatic; with respect to imaging subtypes, 8 had parenchymal hematoma and 1 had intraventricular hemorrhage. Therefore, exploratory analyses separating clinically relevant subtypes were not feasible because of the small number of events in each category. Future studies with larger sample sizes are needed to validate whether early postoperative PI is differentially associated with symptomatic hemorrhage, parenchymal hematoma, or asymptomatic hemorrhagic transformation.

Several additional limitations should be acknowledged. First, this was a single-center retrospective study with a relatively small sample size, and the limited number of hemorrhagic events (n = 17) restricted the number of variables that could be entered into the multivariable model and resulted in wide confidence intervals for the odds ratio estimates. Although Firth penalized regression and Bootstrap internal validation suggested acceptable internal discrimination (optimism-corrected AUC 0.810; calibration slope 1.019, Brier score 0.1045), the small number of events means that definitive model stability cannot be established; therefore, the model should be regarded as exploratory, and the effect estimates should not be overinterpreted. Second, TCD examination is operator-dependent and relies on an adequate temporal bone window, which may limit its applicability in approximately 10%–15% of patients. Although the TCD operator was blinded to subsequent imaging outcomes, intra-observer reliability of PI measurement was not formally assessed. Third, dynamic post-procedural factors such as blood pressure variability and the timing of antithrombotic agents were not captured. In addition, PI is influenced by systemic hemodynamic factors such as blood pressure, heart rate, PaCO₂, hemoglobin, and vascular compliance, which were not systematically collected in this retrospective cohort. Therefore, the association between PI and cerebral hemorrhage may be partially mediated by systemic hemodynamic status or differences in postoperative blood pressure management. Future studies should incorporate these variables to clarify the independent contribution of PI. Fourth, data on prior antiplatelet or anticoagulant use, dyslipidemia, and chronic kidney disease were not systematically collected in this retrospective cohort. These factors may influence the risk of cerebral hemorrhage and represent potential sources of residual confounding. Fifth, the lack of an external validation cohort limits the generalizability of the prediction model. Future multicenter prospective studies are needed to externally validate the model and to determine whether management strategies incorporating early PI assessment can improve clinical outcomes.

## 5 Conclusion

The early postoperative ipsilateral middle cerebral artery PI is a potential prognostic marker for any radiographic intracranial hemorrhage after EVT for anterior circulation large vessel occlusion acute ischemic stroke, with an optimal cutoff of 0.735. Adding PI to diabetes mellitus and ASPECTS was associated with a modest improvement in discrimination (ΔAUC = 0.088, *p* = 0.011) and continuous net reclassification improvement (NRI = 0.594, *p* = 0.020), whereas categorical NRI was not significant. However, because both diabetes mellitus and ASPECTS were selected from the present dataset, this finding should be interpreted only relative to this specific reference model and not as evidence of incremental value beyond all established predictors. Because of the uncertain temporal relationship between PI measurement and hemorrhage onset and the limited number of events, these findings should be regarded as exploratory and require validation in larger cohorts with serial imaging and subtype-specific analyses. No clinical recommendations can be made based on these retrospective findings.

## Data Availability

The original contributions presented in the study are included in the article/supplementary material, further inquiries can be directed to the corresponding authors.

## Glossary

AIS: Acute ischemic stroke
ASPECTS: Alberta stroke program early CT score
AUC: Area under the curve
BBB: Blood–brain barrier
CI: Confidence interval
CTA: Computed tomography angiography
DSA: Digital subtraction angiography
eTICI: Expanded thrombolysis in cerebral infarction
IQR: Interquartile range
MCA: Middle cerebral artery
mRS: Modified Rankin scale
MRA: Magnetic resonance angiography
NIHSS: National institutes of health stroke scale
OR: Odds ratio
PI: Pulsatility index
ROC: Receiver operating characteristic
SD: Standard deviation
TCD: Transcranial Doppler
*Vs*: Systolic peak velocity
Vd: End-diastolic velocity
Vm: Mean velocity
